# Contrasts in Glioblastoma—Venous Thromboembolism versus Bleeding Risk

**DOI:** 10.3390/cells10061414

**Published:** 2021-06-07

**Authors:** Viktoria Muster, Thomas Gary

**Affiliations:** Division of Internal Medicine, Department of Vascular Medicine, Medical University of Graz, 8036 Graz, Austria; Thomas.gary@medunigraz.at

**Keywords:** glioblastoma, venous thromboembolism, bleeding risk, anticoagulation

## Abstract

Glioblastoma is among the tumor entities with an extreme thrombogenic potential and patients are at very high risk of developing a venous thromboembolism (VTE) over the course of the disease, with an incidence of up to 30% per year. Major efforts are currently being made to understand and gain novel insights into the underlying pathomechanisms of the development of VTE in patients with glioblastoma and to find appropriate biomarkers. Yet, patients with glioblastoma not only face a high thromboembolic risk but are also at risk of bleeding events. In the case of VTE, a therapeutic anticoagulation with low molecular weight heparin or, in the case of low bleeding risk, treatment with a direct oral anticoagulant, is recommended, according to recently published guidelines. With respect to an elevated bleeding risk in glioblastoma patients, therapeutic anticoagulation remains challenging in this patient group and prospective data for this vulnerable patient group are scarce, particularly with regard to direct oral anticoagulants.

## 1. Introduction

Cancer patients have a four- to seven-fold increased risk of developing a venous thromboembolism, and there is a close relationship between malignancy, abnormal coagulation parameters and thrombosis [1]. The prevention of thromboembolic events is highly relevant, as venous thromboembolism is a dominant cause of death in patients with cancer and is associated with a poor prognosis [2]. The incidence of cancer-associated thrombosis varies according to the tumor site, yet glioblastoma is among the tumor entities with an extreme thrombogenic potential, and patients are at very high risk of developing a venous thromboembolism (VTE) over the course of the disease, with an incidence of up to 30% per year [3,4,5]. Yet, not only is thrombosis associated with hemostatic disorders in glioblastoma but it is also associated with hemorrhage, which occurs both locally and systemically [6,7,8].

Current research aiming to gain novel insights into the thrombophilic state of patients with glioblastoma is endeavoring to understand its underlying pathomechanisms and how the aggressiveness of the tumor entity is linked to the high incidence of VTE in this patient group.

## 2. Risk Factors and Biomarkers

Molecular as well as laboratory biomarkers in glioblastoma patients with cancer associated VTE are clinically difficult to assess; therefore, it is also unknown to which extend each of the relevant biomarkers (see Table 1) influence the overall thrombotic risk of an individual patient. Yet, it is known that the mechanisms that contribute to tumor progression, which include tumor cell expression of hemostatic proteins, microparticle production, inflammatory cytokines, proangiogenic factors and expression of adhesion molecules, are also among the principal mechanisms of cancer-associated VTE [9]. However, it is not only laboratory and molecular biomarkers that influence VTE risk in glioblastoma patients; treatment-related and patient-related risk factors are also further influences and need to be taken into account when assessing the individual VTE risk in glioblastoma patients.

### 2.1. Tissue Factor and Circulating Microparticles Bearing Tissue Factor

In cancer patients, blood clotting is directly activated by tumor cells through the production and release of procoagulant factors such as tissue factor. The tumor itself releases tissue-factor positive microparticles into the circulatory system, which may contribute to cancer-associated thrombosis [10,11]. Additionally, the tumor cell-derived tissue factor influences the expression of vascular endothelial growth factor in the tumor cells, as well as in the host vascular cells, and regulates the tumor neo-vascularization. This mechanism demonstrates an important link between activation of coagulation, thrombosis and inflammation, as well as tumor growth [9]. Furthermore, high levels of circulating microparticles are found in cancer patients and may contribute to the hypercoagulable state in these patients [12,13]. In contrast to these findings, Thaler et al. reported that only low levels of tissue factor in association with circulating microparticles were found in patients with glioblastoma which, in addition, did not predict the risk of VTE [14].

In mice models, it was found that low levels of tissue factor may result in spontaneous fatal brain hemorrhage. It was also found that the administration of antihuman tissue factor antibodies can result in cerebral hemorrhage. Furthermore, also in generated mice with very low expression levels of tissue factor, increased intracerebral hemorrhage was found in comparison to controls [15,16]. These findings may suggest that high levels of tissue factor in the brain are needed to limit intracerebral hemorrhage after brain injury, such as tumor biopsy or resection, and thus may lead to the very high risk of tumor-associated thrombosis in brain tumors, especially in the postoperative period [11].

### 2.2. Factor VIII

Elevated Factor VIII plasma levels are found in approximately 20% of patients with VTE [17,18]. Additionally, in patients with glioblastoma, elevated factor VIII levels are associated with a 2.1-fold increase of risk of developing a VTE [19].

### 2.3. D-Dimer Levels

Studies on D-dimer levels in patients with glioblastoma reveal different results, with data suggesting that D-dimer levels are not associated with an increase in VTE risk and studies finding an association between high D-dimer levels and the risk of thromboembolic events [19,20,21]. Posch et al. showed in their study of longitudinal measurements that, taking into account that patient-, tumor- and therapy-related risk factors of each patient can change over the course of the disease, elevated D-dimer levels can be found in a specific patient but may remain stable over time when no VTE occurs. In patients who develop VTE, a high increase in D-dimer levels was found. This study included 50 patients with primary brain tumors [22].

### 2.4. Isocitrat Dehydrogenase Mutation Status

A large subset of glioma shows somatic point mutations of the isocitrate dehydrogenase (IDH) [23]. WHO grade IV glioblastomas, the most infiltrative glioma, have a wild-type IDH, whereas the majority of IDH mutant gliomas do not fulfill the criteria for WHO grade IV glioblastoma, suggesting that IDH mutation may lead to the inhibition of the development of diagnostic features for WHO grade IV glioblastoma [24]. Unruh et al. furthermore found in their study that 26% of the IDH-wildtype glioma patients develop a venous thromboembolism and, in contrast, 0% of the patients with mutant IDH glioma developed a venous thromboembolism. Moreover, the overall cumulative incidence of VTE in all grades of glioma was higher in the IDH wild-type patients than for patients with IDH mutation (26.5% versus 8.7%), and VTE in IDH mutant patients mainly occurred in in the case of a low-grade glioma. It was found that the risk of VTE increases three-fold in IDH wild-type compared to IDH mutation. This is also consistent when adjusted for age, tumor grade and presence of hemiparesis, as the risk remains twice as high in patients with IDH wild-type compared to IDH mutation glioma [5]. A link between the IDH mutation status and frequency of VTE could be the podoplanin expression levels because IDH-mutant gliomas suppress the expression of podoplanin [5,25,26,27,28]

Yet, the perioperative measurements of prothrombin and partial thromboplastin times did not show any difference between the IDH wild-type and IDH mutant gliomas, suggesting that clotting factors have no functional differences [24]. Interestingly it was also found that tissue factor microparticle activity was elevated in IDH wild-type gliomas compared to IDH-mutant gliomas, and a positive correlation between the preoperative circulating tissue factor microparticle activity and the development of venous thromboembolism was reported [24]. 

### 2.5. Podoplanin Expression

Podoplanin, a sialomucin-like glycoprotein, is frequently expressed by primary brain tumors and has the ability to induce blood platelet activation through binding to the C-type lectin receptor type 2 [29,30,31]. In patients with glioblastoma, its expression is associated with a worse survival rate [28,32]. In the Vienna Cancer and Thrombosis study it was found that, in high-grade, podoplanin-expressing gliomas, peripheral blood parameters were also significantly affected (lower blood platelet count, higher level of Factor VIII acitivity and higher prothrombin fragment 1 + 2). Furthermore, the risk of VTE in podoplanin expressing high-grade glioma was significantly higher compared to podoplanin-negative high-grade glioma (hazard ratio = 3.44; 95% confidence interval, 1.19–9.95; *p* = 0.022), and podoplanin expression in these patients was also associated with a higher mortality [33]. Furthermore it was found that a high podoplanin expression only occurred in IDH wild-type tumors [34].

### 2.6. O6-Methylguanine-DNA Methyltransferase (MGMT) Promoter Methylation

In glioblastoma patients, Diaz et al. found that the incidence of VTE does not differ with or without MGMT promoter methylation, with a cumulative risk of VTE of 30.8% in patients without methylation versus 32.7% in patients with methylation [5]. This is also consistent with the results found by Lim et al., who also found that MGMT promotor methylation status does not show any difference with regard to the risk of VTE development [35].

### 2.7. Glioblastoma Subtype

The World Health Organization (WHO) classifies diffuse infiltrating gliomas into grades II–IV based on histological features. In glioblastoma WHO grade IV, the most aggressive subtype, the incidence of VTE is up to 30% per year compared to 9.2% in WHO grade III, and 8.2% in WHO grade II glioma [3,4,5].

Furthermore, different molecular subtypes of glioblastoma, such as proneural, neural, classical and mesechymal, respond differently to aggressive therapy [36,37]. Yet, up to this point, there are no data available to suggest whether a certain molecular subtype is associated with a higher risk of VTE. As such, future studies are needed. 

### 2.8. Patient Related Risk Factors

Advanced age is a well-known patient-related risk factor for the development of VTE in cancer patients [38,39,40]. Furthermore, patient age also increases with tumor grade in gliomas from II to IV, with a median age of 42.7, 50 and 61.3 years, respectively [5]. Another patient related risk factor which needs to be taken into account is the sex of the patient. In a study population of newly diagnosed high grade glioma and a median follow-up of two years, 17% of the patients developed VTE—79.2% male versus 20.8% female [41].

Patients with glioblastoma and of advanced age are known to have a high risk of venous thromboembolism compared to patients with gliomas of grades II–III. The data of Unruh et al. suggest that this may be due to less common IDH mutations in older patients with glioblastoma [24]. Other patient-related risk factors for the development of VTE in cancer patients are medical comorbidities such as hepatic and renal disease, anemia, previous arterial thromboembolism, congestive heart disease and infection, as well as obesity and the presence of leg paresis [38,39,40]. Previous VTE and the presence of varicose veins also increase the risk of VTE in these patients [42,43].

In a retrospective study, the ABO blood group was discussed as a risk factor for the development of VTE, suggesting the highest risk for blood group A and AB in comparison to blood group 0 [44]. Yet, in a prospective study conducted by the same authors, these findings could not be confirmed [19].

### 2.9. Treatment-Related Risk Factors

The definite diagnosis and grading of gliomas require the extraction of tumor tissue. In the RIETE registry, recent surgery was associated with a higher risk of VTE in glioblastoma patients compared to other cancer types (36% versus 14%) [45]. Yet, it was also found that the frequency of VTE in high-grade glioma patients is also influenced by the surgical approach, with higher rates of VTE found postoperatively after biopsy and subtotal tumor resection compared to high-grade glioma patients with total tumor resection [46]. These data are consistent with the data published by Streiff et al. where it was also found that patients treated with a bioptical approach had a three-fold higher risk of developing VTE [19]. Further treatment-related risk factors in glioblastoma patients are the use of chemotherapy and a longer duration of surgery > 4 h [38]. A co-medication with corticosteroids at the time of VTE diagnosis is found in a very high percentage of patients with glioblastoma (70% versus 13%) [45].

## 3. Bleeding Risk

Bearing in mind the high risk of venous thromboembolism, a general administration of a medical thromboprophylaxis is not recommended, as intracranial hemorrhage (ICH) is also observed in patients with primary brain tumors without the administration of anticoagulation [47,48]. It was found that, through vascular endothelial growth factor and matrix metalloproteinase, which represent angiogenesis mediators, the tumor itself has the ability to induce ICH, but more data are needed to fully understand the role of these mediators and the pathogenesis of cancer-associated ICH [49]. Furthermore, there are only limited data available investigating the bleeding risk of glioblastoma patients receiving anticoagulation, resulting in a high degree of uncertainty for clinicians in relation to the best management of each individual patient. 

In several retrospective studies, the relationship between anticoagulation for VTE and the risk of ICH in glioblastoma patients was investigated, and an ICH rate between 0% and 12% has been reported [4,50,51,52]. 

Although a neurosurgical intervention was not needed for the majority of the patients with ICH, Khoury et al. report that 15.5% of patients with glioblastoma and receiving anticoagulation for VTE developed an ICH, whereas only 2.6% of the glioblastoma patients not receiving anticoagulation for VTE developed an ICH. Yet, a better overall survival, as well as a post-VTE diagnosis survival, were observed in glioblastoma patients with VTE who were receiving anticoagulation. This may partially be explained by a selection bias in treatment, as patients in massively reduced general condition may not have received an anticoagulation. A correlation between ICH and type of anticoagulation was not found in this study. Furthermore, a correlation between the presence or size of tumor, as well as extent of surgical resection and incidence of ICH, were likewise not observed, and thus should not be a contraindication for anticoagulation [53]. This is also consistent with the results of a meta-analysis showing that the overall risk for ICH in glioblastoma patients is more than three-fold higher during anticoagulation treatment compared to not receiving anticoagulation [54].

Regarding the correlation between ICH and the receipt of an anticoagulation agent in high-grade glioma, Mantia et al. performed a retrospective analysis in patients with WHO grade III or IV glioma and who were receiving a therapeutic anticoagulation with enoxaparin for the treatment of VTE. A three-fold increase of major ICH at 1 year was found in the enoxaparin group [55]. 

New therapeutic options in cancer-associated VTE arose when the results of the studies investigating the direct oral antagonists edoxaban, apixaban and rivaroxaban in a head-to-head analysis with low molecular weight heparin were presented. The data from these studies point out a non-inferiority of the direct oral antagonists for the treatment of cancer-associated VTE compared to dalteparin with the endpoint of VTE recurrence, but an increase in gastrointestinal and genitourinary bleeding was found in the group of direct oral anticoagulants. Yet, only a small number of patients with primary brain tumor were included in these studies or were excluded, as in the Caravaggio trial. Therefore, the question of whether direct oral anticoagulants increase the risk of ICH in patients with primary brain tumor currently remains unanswered [56,57,58,59] (Table 2). 

In a retrospective study by Carney et al., it was found that brain tumor patients receiving direct oral anticoagulants (apixaban or rivaroxaban) for the treatment of cancer-associated VTE had a cumulative incidence of ICH at 12 months of 0% versus 36.8% in patients treated with enoxaparin. The post-ICH 30-day case–fatality mortality was 39% in the enoxaparin group and 0% in the direct oral anticoagulant group. Yet, the retrospective study design and possible patient selection bias for the treatment (patients with a high bleeding risk were more likely to receive low molecular weight heparin) limit the findings of this study. Nevertheless, a treatment with direct oral anticoagulants seems to be a possible option in case of cancer-associated VTE in these patients [60].

## 4. Thromboprophylaxis and Therapy According to Current Guidelines

### 4.1. Thromboprophylaxis

The risk–benefit ratio of several prophylactic VTE measures for patients with primary brain tumor undergoing craniotomy was analyzed in a meta-analysis including 1263 patients. Prophylactic VTE measures did not significantly increase the bleeding risk, yet led to significantly lower risk of VTE. The strongest risk reduction for VTE was found in patients receiving unfractionated heparin compared to patients receiving placebo (RR = 0.27; 95% CI 0.1–0.73). Additionally, low molecular weight heparin combined with mechanical prophylaxis resulted in a lower VTE risk than mechanical prophylaxis alone (RR = 0.61; 95% CI 0.46–0.82) [61].

In the AVERT study, apixaban was compared with placebo for the long-term prophylaxis of VTE in cancer patients. Patients with a brain tumor accounted for 4.8% of the patients receiving apixaban compared to 3.5% of the patients receiving placebo. Although a significant reduction in VTE in the apixaban group was found in this study, further conclusions for patients with primary brain tumors cannot be drawn due to the small number of patients with this tumor entity included [62].

Current guidelines from the international society on thrombosis and hemostasis, as well as the 2019 updated international clinical practice guidelines, do not recommend medical prophylaxis for outpatients with brain tumors not undergoing neurosurgery [63,64].

For patients with primary brain tumors undergoing neurosurgery, the use of low molecular weight heparin or unfractionated heparin is recommended, and should be commenced postoperatively and continued until discharge [63]. Additionally, a mechanical thromboprophylaxis with intermittent pneumatic compression can be initiated preoperatively [65].

### 4.2. Therapy

Driven by the publication of the results of the randomized controlled trials comparing low molecular weight heparin and direct oral anticoagulant with regard to effectiveness and safety, current guidelines have been updated and reflect these results in their recommendations. Although one can find minimal differences in the various guidelines, there is a general consensus that direct oral anticoagulants can be used for the initial treatment of cancer-associated VTE in the absence of a high gastrointestinal and/or genitourinary bleeding risk. Otherwise, anticoagulation should be established with low molecular weight heparin [63,66].

Regarding the risk–benefit ratio in patients with a primary tumor, the best treatment option remains uncertain due to limited data. The 2019 international clinical practice guidelines for the treatment and prophylaxis of venous thromboembolism in patients with cancer recommend low molecular weight heparin or direct oral anticoagulants for the treatment of established VTE in patients with a brain tumor (grade 2B recommendation) [63]. In accordance with the guidelines of the American Society of Clinical Oncology, anticoagulation should be offered to patients with a primary brain tumor and VTE. Yet, uncertainty remains regarding the choice of agent and only limited safety data are available regarding direct oral anticoagulants in this patient group [66].

## 5. Conclusions

Patients with glioblastoma are at a high risk of developing VTE over the course of the disease. Great efforts are being made to better understand the underlying pathomechanisms and risk factors for the thrombophilic situation, as well as the bleeding risk in these patients, highlighting the patients with the greatest risk with the use of biomarkers, for instance. A high diversitiy in patient and biomolecular characteristics, as well as different treatment options, raise the need for future studies to enable a more personalized approach for this patient group. Furthermore, there are only limited data available investigating the bleeding risk of glioblastoma patients receiving anticoagulation, leading to a high degree of uncertainty for clinicians in terms of the best management of each individual patient while taking into account that, despite the high bleeding risk in these patients, anticoagulation may yet be beneficial regarding life expectancy and quality of life.

## Figures and Tables

**Table 1:** Risk factors for venous thromboembolism in glioblastoma patients.

Risk Factors for Venous Thromboembolism in Glioblastoma Patients
Biomarkers
tissue factor and circulating microparticles bearing tissue factor
coagulation Factor VIII
D-dimer levels
Tumor related
IDH-mutation status
podoplanin expression
glioblastoma subtype
Patient related
leg paresis
advanced age
obesity
medical comorbidities
varicose veins
previous VTE
Treatment related
biopsy and subtotal tumor resection
duration of surgery > 4 h
corticosteroid co-medication
chemotherapy

Abbreviations: IDH = Isocitrat dehydrogenase; VTE = venous thromboembolism.

**Table 2:** Randomized controlled trials, including patients with primary brain tumor or brain metastasis, on safety and efficacy of direct oral anticoagulants compared to low-molecular-weight heparin in the treatment of cancer-associated venous thromboembolism.

Trial [Ref.]	Hokusai VTE-Cancer Study [56]	SELECT-D Study [57]	Adam-VTE Study [58]	Caravaggio Trial [59]
DOAC investigated	Edoxaban	Rivaroxaban	Apixaban	Apixaban
Brain tumor or Brain metastasis Patients included *n*	74	3	8	x

Abbreviations: Ref = reference; DOAC = direct oral anticoagulant; VTE = venous thromboembolism.
